# Spatiotemporal Control of Doxorubicin Delivery from “Stealth-Like” Prodrug Micelles

**DOI:** 10.3390/ijms18102033

**Published:** 2017-09-22

**Authors:** Li Kong, Dimitrios Poulcharidis, Gregory F. Schneider, Frederick Campbell, Alexander Kros

**Affiliations:** Supramolecular and Biomaterials Chemistry, Leiden Institute of Chemistry, Einsteinweg 55, 2333 CC Leiden, The Netherlands; l.kong@chem.leidenuniv.nl (L.K.); d.poulcharidis@chem.leidenuniv.nl (D.P.); g.f.schneider@chem.leidenuniv.nl (G.F.S.)

**Keywords:** micelles, prodrugs, doxorubicin, light activation

## Abstract

In the treatment of cancer, targeting of anticancer drugs to the tumor microenvironment is highly desirable. Not only does this imply accurate tumor targeting but also minimal drug release en route to the tumor and maximal drug release once there. Here we describe high-loading, “stealth-like” doxorubicin micelles as a pro-drug delivery system, which upon light activation, leads to burst-like doxorbicin release. Through this approach, we show precise spatiotemporal control of doxorubicin delivery to cells in vitro.

## 1. Introduction

Doxorubicin (DOX) is a potent cytotoxic drug used in the clinical treatment of many human cancers. Administered alone, and with no inherent cell selectivity, the clinical efficacy of DOX is however hampered by off-target cardiotoxicity [1]. This limits the cumulative patient lifetime dose of DOX to just 550 mg/m^2^, irrespective of therapeutic success [2]. Considerable efforts have been made to improve the therapeutic index of DOX by localizing its extracellular release to the tumor microenvironment alone. Typically, this involves chemical modification or vector entrapment of DOX (e.g., within long-circulating liposomes). Within these systems, strategies to enhance tumor targeting and/or local DOX release include the use of active targeting ligands [3], steric shielding (e.g., PEGylation) of DOX carriers [4], exploitation of endogenous (e.g., low pH within the tumor environment) and exogenous (e.g., heat, magnetism, ultrasound or light) stimuli [5], and combinations thereof [6].

Of these various approaches, the passive targeting of liposome-entrapped DOX to tumors remains the only strategy approved for clinical use. Liposomal-DOX formulations (e.g., Myocet^®^, Doxil^®^) are used to treat a variety of malignant human cancers, including select breast and ovarian cancers, multiple myeloma and AIDS-related Kaposi’s sarcoma. These liposome formulations, optimally 100 nm in size and administered systemically, are designed to passively accumulate within the tumor via the enhanced permeability and retention (EPR) effect. This phenomena is characterized by the ill-defined (“leaky”) vasculature and poor lymphatic drainage of many tumor pathologies [7]. Through this approach high local concentrations of DOX are achieved within the tumor following prolonged and passive drug leakage across the liposome membrane. For this strategy to be effective, liposomes with long circulation lifetimes are sought. Care must therefore be taken to balance the conflicting need to both minimize DOX leakage en route to the tumor while ensuring therapeutically relevant concentrations are released once there. Drug retention and release profiles can be fine-tuned through judicious choice of drug-to-lipid ratios and liposome lipid composition, and circulation lifetimes can be increased through steric shielding (typically PEGylation) of the liposome surface (to create “stealth” liposomes) [4], however finding the necessary balance between drug retention and release is an intrinsic limitation of these nanoparticle systems [8].

**Figure 1:**
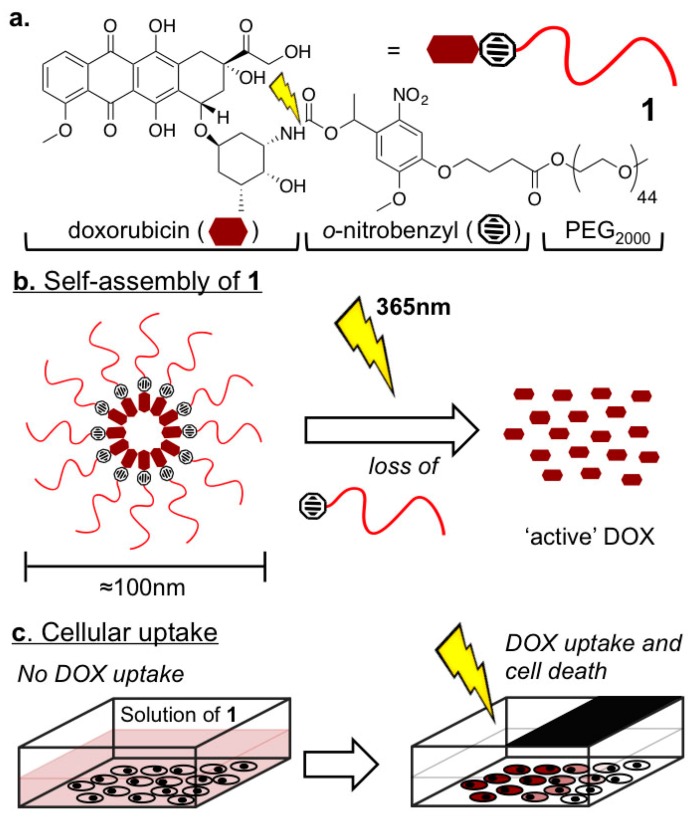
Light activated doxorubicin pro-drug micelles. (**a**) Doxorubicin-*ortho*-nitrobenzyl-mPEG_2000_ construct, **1**; (**b**) Self-assembly of 1 in aqueous media to 100 nm PEGylated and DOX (doxorubicin)-rich micelles from which quantitative drug release is triggered by light; (**c**) Light directed DOX release, cell uptake, and cell death.

In this communication, we describe light activated, DOX-rich (20 wt % drug loading) micelles, which prior to light activation, share analogous physicochemical properties (size, morphology, surface chemistry) to those of long circulating liposomal-DOX formulations. Crucially however we observe no premature DOX release (and therefore cytotoxicity) in the absence of light. Upon light activation, quantitative drug release is achieved (Figure 1). These properties represent a significant technological improvement over analogous DOX-PEG prodrug systems triggered by tumor-specific, endogenous stimuli (pH [9,10,11,12], reduction [13], enzymatic [14]), for which DOX release is typically slow (hours) and incomplete, as well as those reliant on external stimulus, such as light [15,16,17,18,19], for which reported physicochemical properties (size, morphology, surface chemistry) preclude long circulation lifetimes necessary for efficient tumor accumulation via the EPR effect.

## 2. Results

### 2.1. Biophysical Characterization of Light-Activated DOX-PEG Prodrug Micelles

**Figure 2:**
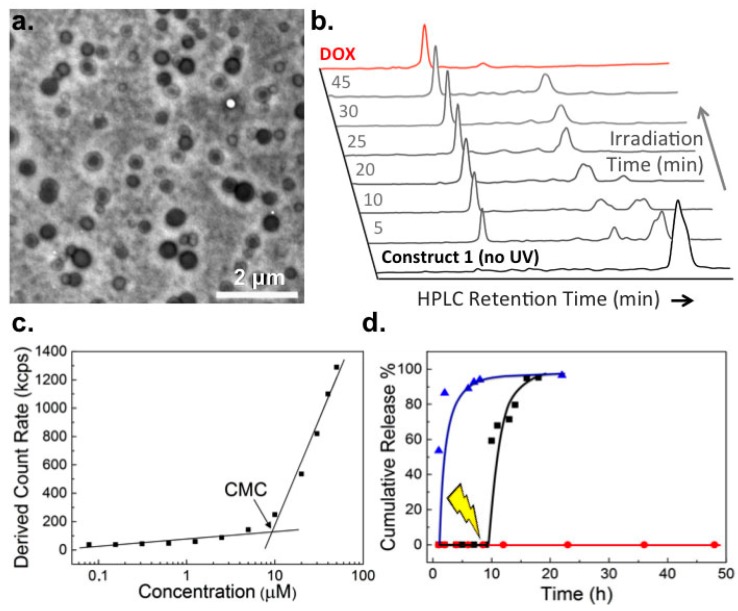
Characterization of doxorubicin pro-drug micelles and light induced drug release. (**a**) TEM (transmission electron microscopy) image (uranyl acetate stain) of micelles of **1** (300 μM, approx. 0.7 mg/mL); (**b**) Time evolution of the HPLC (high-performance liquid chromatography) spectra of a solution of **1** (100 μM in PBS (phosphate buffered saline)) during photolysis (365 nm, 3–5 mW/cm^2^). Free DOX (100 μM), dissolved in PBS, was used to confirm clean photolysis of **1** to release “active” DOX. HPLC conditions described in Materials and Methods; (**c**) CMC (critical micelle concentration) determination by light scattering following serial dilution of **1** (100 μM–75 nM) in PBS; (**d**) In vitro DOX release profiles from **1** (300 μM) in PBS. No UV irradiation (**red**), UV irradiation at 9 h (**black**) and free DOX control (**blue**).

The synthesis and characterisation of photoactivatable DOX-*ortho*-nitrobenzyl-PEG construct, **1**, is described in the Supplementary Information (Figures S1–S6). Self-assembly of **1** in aqueous media resulted in particles with mean hydrodynamic diameters of 100 nm and ranging in size from 30 to 300 nm (PDI 0.25, Figure S7 in Supplementary Materials). TEM (transmission electron microscopy) measurements revealed “loose” core–shell micelle structures in which the nanoparticle core appears electron-rich (high contrast) and likely contains DOX (Figure 2a). Similar morphologies have been reported for analogous DOX-PEG assemblies [11,12,14]. The critical micelle concentration (CMC) of self-assembled micelles of **1** was determined to be 9.2 µM (approx. 25 µg/mL, Figure 2b) and particles were stable over time, over a range of concentrations and diluted in complete cell culture media (Figure S7 in Supplementary Materials). Upon low power UV irradiation (365 nm, 3–5 mW/cm^2^), complete photolysis of self-assembled **1** to pharmacologically “active” DOX was achieved within 25 min, however significant DOX release was observed following just 5 min low-power UV irradiation (Figure 2c). Drug release was quantitative and importantly, no premature leakage of DOX was observed in the absence of light activation (Figure 2d).

### 2.2. In Vitro Toxicity

**Figure 3:**
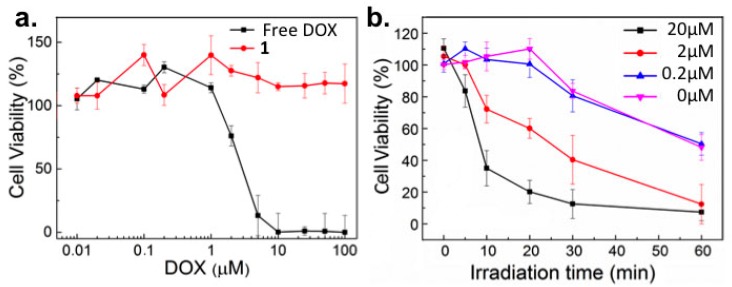
Viability of HeLa cells in vitro treated with doxorubicin pro-drug micelles. (**a**) Cell viability following incubation with varying concentrations (10 nM–100 μM) of free DOX (**black**) and **1** (**red**) in the absence of light; (**b**) Viability of HeLa cells in vitro treated with varying concentrations of **1** and irradiated (365 nm, 15–17 mW/cm^2^) for up to 1h. Pink line corresponds to photoinduced cytotoxicity.

Next, the cytotoxicity of **1** was assessed against cancer (HeLa) cells in vitro. While the measured IC_50_ value of free DOX was 3 μM, **1** showed no cytotoxic effect up to the highest concentration tested (100 μM) in the absence of light (Figure 3a). Upon light activation (365 nm, 15–17 mW/cm^2^) however, DOX induced cytotoxicity correlated, as expected, with both increased concentrations of **1** as well as increasing irradiation time (Figure 3b).

Importantly, UV-A light induced cytotoxicity (due to UV-A induced oxidative stress) [20], only resulted in significant cell death following >30 min continuous irradiation (Figure 3b, pink line, and Figure S8 in Supplementary Information). This is significantly longer than the irradiation time required to release effective concentrations of DOX (released from 20 µM solutions of **1**) achieving >50% cell death. It is also important to note, below its CMC (9.2 µM), the cytotoxicity of **1** was also insignificant. While this is likely due to the membrane impermeability of individual DOX-PEG constructs, these systems will no longer exist as nanoparticle assemblies and will likely demonstrate very different in vivo pharmacokinetic profiles (i.e., low vascular retention, rapid renal filtration) compared to 100 nm micelles of **1** [21].

### 2.3. Light-Templated Cellular Uptake of DOX

**Figure 4:**
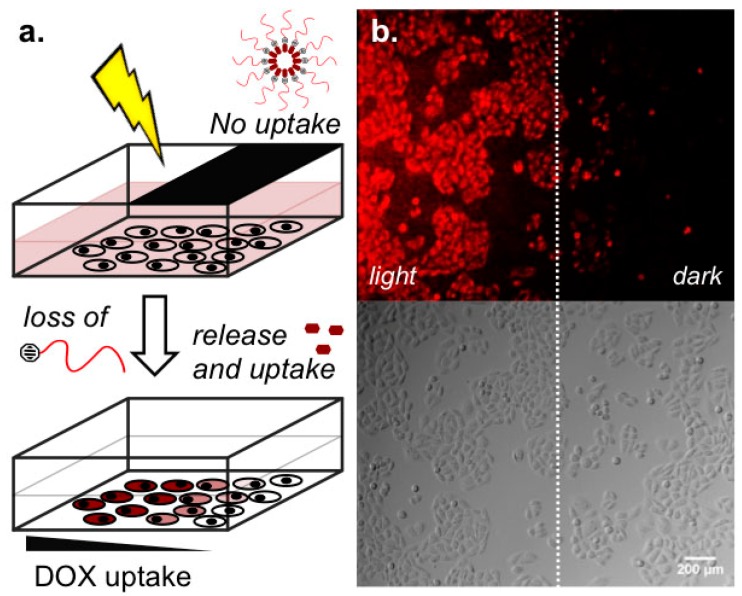
Light templated doxorubicin delivery in vitro. Patterned light (365 nm, 15–17 mW/cm^2^) activation of **1** (300 μM) and cellular uptake of DOX (**red**).

Increasing DOX cellular uptake with increasing time of light activation of **1** was confirmed by FACS analysis (Figure S9 in Supplementary Information) and to demonstrate the precision afforded by the described DOX delivery prodrug system, micelles of **1** were first incubated with cells then UV light applied over just half the well plate (Figure 4a). The result was clear spatial delineation of DOX cellular uptake in vitro (Figure 4b), highlighting not only efficient photolysis of **1** but also rapid cellular uptake of DOX once released.

## 3. Discussion

Here we demonstrate rapid and quantitative release of DOX from self-assembled micelles of **1** triggered by light. Prior to light activation, DOX-rich micelles are not cytotoxicity, do not release DOX prematurely and share near identical physicochemical character to that of marketed and long-circulating liposome-DOX formulations (e.g., Doxil^®^). Towards tumor targeting of DOX in vivo, it is envisaged micelles of **1**, administered systemically, will first passively accumulate within the tumor microenvironment via the EPR effect whereupon drug release could be triggered by light, on demand. Given the limited tissue penetration of single photon UV light, options to apply UV light to tumors residing deep within the body include the use of fiber-optic endoscopic techniques [22] or 2-photon light activation [23]. Alternatively, strategies rendering this system sensitive to longer wavelength, single photon, near-infrared (NIR) light can be considered [24]. Future studies will focus on the application of these micelles in vivo and their potential use as an anti-cancer drug delivery system. In particular, care must be taken to maintain the concentration of **1** above the CMC following dilution in blood (approximately 5 L for an adult human) [25]. For the system described, this equates to an injected dose of >130 mg/5L of **1**—approximately 30 mg DOX. This figure is below the FDA recommended dosage for DOX·HCl (40–60 mg/m^2^ administered every 21–28 days) currently used in the treatment of a wide range of human cancers.

## 4. Materials and Methods

### 4.1. Materials and Instruments

Doxorubicin hydrochloride (DOX·HCl) was purchased from Cayman Chemical Company (Ann Arbor, MI, USA) and used without further purification. All other chemical reagents were purchased from Sigma-Aldrich (Zwijndrecht, The Netherlands) and used without further purification. All solvents were purchased from Biosolve Ltd. (Valkenswaard, The Netherlands). Phosphate buffered saline (PBS): 5 mM KH_2_PO_4_, 15 mM K_2_HPO_4_, 150 mM NaCl, pH 7.4. Silica gel column chromatography was performed using silica gel grade 40–63 μm (Merck & Co., Amsterdam, The Netherlands). TLC analysis was performed using aluminum-backed silica gel TLC plates (60_F_ 254, Merck, Amsterdam, The Netherlands), visualization by UV absorption at 254 nm and/or staining with KMnO_4_ solution. NMR (nuclear magnetic resonance) spectra were measured on a AV-400 MHz spectrometer (Bruker Nederland BV, Leiderdorp, MA, USA). Chemical shifts are recorded in ppm. Tetramethylsilane (TMS) is used as an internal standard. Coupling constants are given in Hz. LCMS analysis was performed on a Nanoacquity UPLC system-Synapt G2Si mass spectrometer (Waters Corporation, Milford, MA, USA) operating MassLynx software. Separation (Acquity UPLC M-Class 300 µm × 50 mm column, packed with BEH C4 material of 1.7 µm diameter and 300 Å pore size particles, flow rate: 2 µL/min; Waters Corporation, Milford, MA, USA) was carried out over a linear gradient of 10–90% **B** over 20 min. Buffers: **A**—H_2_O (0.1% Formic Acid); **B**—Acetonitrile (0.1% Formic Acid). Electro-spray ionization (ESI) via Nano-spray source with ESI emitters (New Objective Inc., Woburn, MA, USA) fused silica tubing 360 µm OD × 25 µm ID tapered to 5 ± 0.5 µm (5 nL/cm void volume). MS (mass spectrometry) settings (positive resolution mode): source temperature of 80 °C, capillary voltage 4.5 kV, nano flow gas of 0.25 Bar, purge gas 250 L/h, trap gas flow 2.0 mL/min, cone gas 100 L/h, sampling cone 25 V, source offset 25, trap CE 32 V, scan time 3.0 s, mass range 400–2400 *m*/*z*. Lock mass acquiring was done with a mixture of Leu-Enkephalin (556.2771) and [Glu1]-fibrinopeptide B (785.84265), lockspray voltage 3.5 kV, [Glu1]-fibrinopeptide B fragmentation was used as calibrant. MaxEnt 1 was used for mass deconvolution of the envelopes (Cambridge, UK). HPLC (high-performance liquid chromatography) analysis was performed using a Shimadzu HPLC setup equipped with two LC-8A series pumps (Shimadzu Europa GmbH, ‘s-Hertogenbosch, The Netherlands). Separation: Prep (Kinetex EVO, C18 column, 5 μm, 150 × 21.2 mm, flow rate: 15 mL/min; Phenomenex B.V., Utrecht, The Netherlands), analytical (Vision HT, C18 column, 5 μm, 150 × 4.6 mm, flow rate: 1 mL/min; Phenomenex B.V., Utrecht, The Netherlands), in all instances, was carried out over a linear gradient of 10–95% **B** over 25 min with an initial 5 min hold at 10% **B**. HPLC buffers: **A**—H_2_O (0.1% TFA); **B**—Acetonitrile (0.1% TFA). UV detection at 254 nm.

For experiments not involving cells, UV light irradiation was performed using a hand-held BLAK-RAY B-100AP high intensity UV lamp (365 nm, 100 W; Fisher Scientific, Hampton, NH, USA) encased in a cardboard box. Samples were irradiated in quartz cuvettes at a fixed distance of 10 cm from the UV source. For all cell experiments, UV light irradiation was performed using a high-power LED (365 nm, Roithner Laser Technik GmbH, Vienna, Austria) mounted at a fixed distance of 1 cm above the cells.

### 4.2. Preperation and Characterization of Light-Activated DOX-PEG Prodrug Micelles

Micelles of **1** were prepared via thin film hydration followed by sonication. Bath sonication (Branson 2510 Ultrasonic Cleaner, Branson Ultrasonics, Danbury, CT, USA) was carried out at 50 °C for 5 min. Particle size distributions were determined using a Malvern Zetasizer Nano ZS (Malvern Instruments Ltd., Malvern, UK) equipped with a peltier controlled thermostatic holder, a fixed wavelength at 633 nm and scattering angle of 173°. DLS measurements were carried out at room temperature. For TEM observation, a drop of **1** (300 μM) was placed onto a nitrocellulose membrane covered TEM copper grid and dabbed dry through the underside of the grid with a tissue. This was then washed three times with ddH_2_O. A drop of uranyl acetate (2% *w*/*v*) in H_2_O was then added and the sample left to dry in the dark. Transmission electron microscopy (TEM JEOL 1010; JEOL Ltd., Tokyo, Japan; Nieuw-Vennep, The Netherlands) was run at an accelerating voltage of 60 kV.

### 4.3. In Vitro Drug Release

To monitor the release profile of DOX following light irradiation, 1mL of **1** (300 µM, >CMC) in PBS were placed in dialysis tubing (MWCO: 3.5 KDa) and dialyzed against 10mL of dialysis buffers (PBS + 0.5% (*w*/*w*) Tween 80). At various time intervals, 3.0 mL of dialysis buffer was removed and replaced with fresh buffer. The amount of free DOX was quantified by UV–Vis absorbance measurements at 480 nm. To monitor light activated release of DOX, a sample of **1** was removed from the dialysis tubing at 9 h and irradiated for 30 min. This solution was returned to the dialysis tubing and the experiment continued. As a positive control, free DOX (300 µM) in PBS was subjected to the identical experimental conditions.

### 4.4. WST Cell Proliferation Assay

HeLa cells were seeded in 96-well plates at a density of 10,000 cells per well and incubated overnight. Cells were washed once with PBS, then micelles of **1** (100 μL, varying concentrations in 1:1 PBS:DMEM + FCS), free DOX solutions (100 μL varying concentrations in 1:1 PBS:DMEM + FCS) or DMEM + FCS alone (100 μL) were added and the cells incubated for 12 h. Cells were then washed three times (DMEM + FCS), fresh DMEM + FCS added and incubated for a further 24 h. Cell media was removed and 200 μL Cell Proliferation Reagent; WST-1 (Sigma Aldrich, Zwijndrecht, The Netherlands) added to each well. Cells were incubated (37 °C) for a further 3 h, according to the supplier guidelines. To determine cell viability, absorbance at 450 nm was measured. All experiments were carried out in quadruplicate. 

### 4.5. FACS Analysis

HeLa cells were incubated with **1** (300 μM in PBS, >CMC) for 30 min then irradiated (365 nm, 15–17 mW/cm^2^) for 15 min. Following irradiation, the solution was carefully removed, cells washed with PBS, trypsinized and immediately analyzed by flow cytometery. Counting and characterization was performed by measuring 10,000 events in triplicate and concatenation of this data. For manual gating, the outermost ring of the dot plot was selected. Quadrants were manually selected to illustrate fluorescence plots. No compensation was required.

### 4.6. Light Templated DOX Devlivery to Cells

HeLa cells were seeded in 24-well plates at a density of 10^5^ cells per mL (6 × 10^4^ cells per well) and incubated overnight. Cells were washed once with PBS, then micelles of **1** (300 μM in PBS, >CMC) added and incubated for 30 min. Next, half of the well was covered with aluminum foil followed by UV irradiation (365 nm, 15–17 mW/cm^2^) from above for 15 min. Following irradiation, the solution was carefully removed, cells washed (3 × DMEM + FCS) and immediately analyzed under the fluorescence microscope.

## Supplementary Materials

Supplementary materials can be found at www.mdpi.com/1422-0067/18/10/2033/s1.
