# Supplementary material for: Spatiotemporal Control of Doxorubicin Delivery from “Stealth-Like” Prodrug Micelles
Source: Int J Mol Sci. 2017 Sep 22;18(10):2033. doi: 10.3390/ijms18102033 (PMC5666715; doi:10.3390/ijms18102033)
Supplement: Supplementary file 1 [file ijms-18-02033-s001.pdf]

Supporting Information

Spatiotemporal control of doxorubicin delivery from ‘stealth’-like prodrug micelles

Li Kong, Dimitrios Poulcharidis, Gregory F Schneider, Frederick Campbell and Alexander Kros**

Contents

Synthesis of 1

Figure S1 – ^{1}H NMR of **3**
Figure S2 – ^{1}H NMR of Doxorubicin
Figure S3 – ^{1}H NMR of **1**
Figure S4 – Analytical HPLC of **1**.
Figure S5 – ESI-MS (raw) spectra following photolysis of **1**.
Figure S6 – Deconvoluted mass spectra of nitroso-PEG envelope
Figure S7 – DLS size distributions of **1** over time and at varying concentrations.
Figure S8 – Bright-field images of cells following varying UV irradiation time.
Figure S9 – FACS analysis of DOX uptake varying UV irradiation time

References

Synthesis of 1

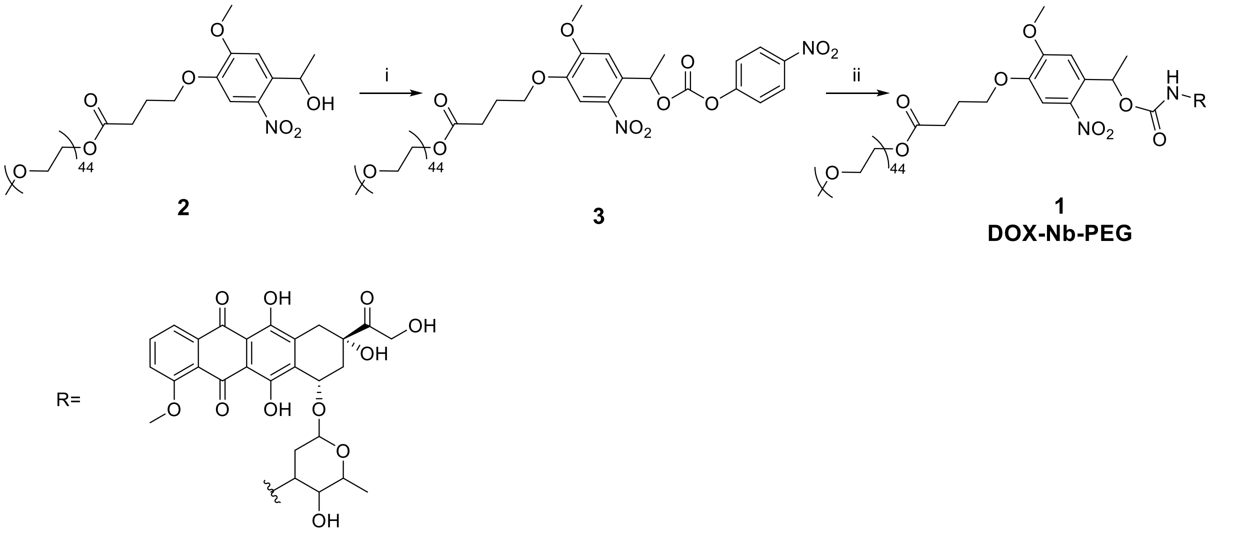

Synthetic scheme to 1. (i) 4-nitrophenylchloroformate, Et_3N, CH_2Cl_2. (ii) Doxorubicin.HCl, Et_3N, DMF.

MethoxyPEG$_{2000}$ 4-(4-(1-hydroxyethyl)-2-methoxy-5-nitrophenoxy)butanoate (2) was synthesized as previously reported [1].

MethoxyPEG$_{2000}$ 4-(2-methoxy-5-nitro-4-(1-(((4-nitrophenoxy)carbonyl)oxy)ethyl) phenoxy)butanoate, 3

To a stirred solution of **2** (500 mg, 0.22 mmol) and 4-nitrophenyl chloroformate (265 mg, 1.31 mmol, 6eq.) in CH_2Cl_2 (20mL) was added Et_3N (305 μL, 2.19 mmol, 10eq.). The reaction mixture was stirred at room temperature in the dark overnight. Following solvent removal *in vacuo*, purification by column chromatography (*Gradient*: CH_2Cl_2 to 15% MeOH in CH_2Cl_2) afforded **3** (278 mg, 0.11 mmol, 52%) as a yellow powder.

R_f: 0.30 (CH_2Cl_2:MeOH; 12:1)

^{1}H NMR ($CDCl_3$, 400 MHz): 8.26 (d, *J = 8 Hz*, Ar*H*–o-NO_2, 2H); 7.61 (s, Ar*H*–*o*-NO_2, 1H); 7.35 (d, *J = 8Hz*, Ar*H*–m-NO_2, 2H); 7.11 (s, Ar*H*–m-NO_2, 1H); 6.52 (q, *J = 8 Hz*, C*H*(CH_3)OCOO, 1H); 4.26 (m,COOC$H_2$$CH_2$O, 2H); 4.14 (t, *J = 8 Hz*, OOC$CH_2$$CH_2CH_2$O, 2H); 4.00 (s, C$H_3$O, 3H); 3.45-3.95 (m, O$CH_2$$CH_2$, 196H); 3.32 (s, CH_3O$CH_2$$CH_2$O,3H); 2.59 (m, OOCC$H_2$$CH_2$$CH_2$O, 2H); 2.19 (m, OOC$CH_2CH_2$$CH_2$O, 2H); 1.78 (d, *J = 8 Hz*, CH(CH_3) OCOO, 3H).

MethoxyPEG$_{2000}$ 4-(4-(1-(((3-hydroxy-2-methyl-6-(((1S,3S)-3,5,12-trihydroxy-3-(2-hydroxyacetyl)-10-methoxy-6,11-dioxo-1,2,3,4,6,11-hexahydrotetracen-1-yl)oxy)tetrahydro-2H-pyran-4-yl)carbamoyl)oxy)ethyl)-2-methoxy-5-nitrophenoxy)butanoate, 1

To a stirred solution of **3** (86 mg, 0.034 mmol) and doxorubicin.HCl (20 mg, 0.037 μmol) in DMF (500 μL) was added Et_3N (47.2 μL, 0.34 mmol, 10eq.). The reaction mixture was stirred at RT in the dark overnight. CH_2Cl_2 (20 mL) was then added to the reaction mixture and the solution washed with brine (15 mL). The organic fraction was dried (Na_2SO_4) and solvent removed *in vacuo*. Column chromatography (*Gradient*: CH_2Cl_2 to 2% MeOH in CH_2Cl_2 to 10% MeOH in CH_2Cl_2) yielded **1** (58.1 mg, 61%) as a red powder.

R_f: 0.20 (CH_2Cl_2:MeOH; 12:1)
^{1}H NMR ($CDCl_3$, 400 MHz): Partial peak assignment annotated in Figure S3. ^{1}H NMR of DOX with partial peak assignment included in Figure S2.
MS – despite numerous attempts to characterize this compound (MALDI, ESI), MS data was inconclusive – most likely due to compound instability and/or poor ionization of this compound during mass spec analysis. Following UV irradiation however, the MS of the photolysis products could be clearly detected (Figure S5 and S6). These products – nitroso-PEG and DOX – can only arise from the photolysis of **1**.

Supporting Information Figures

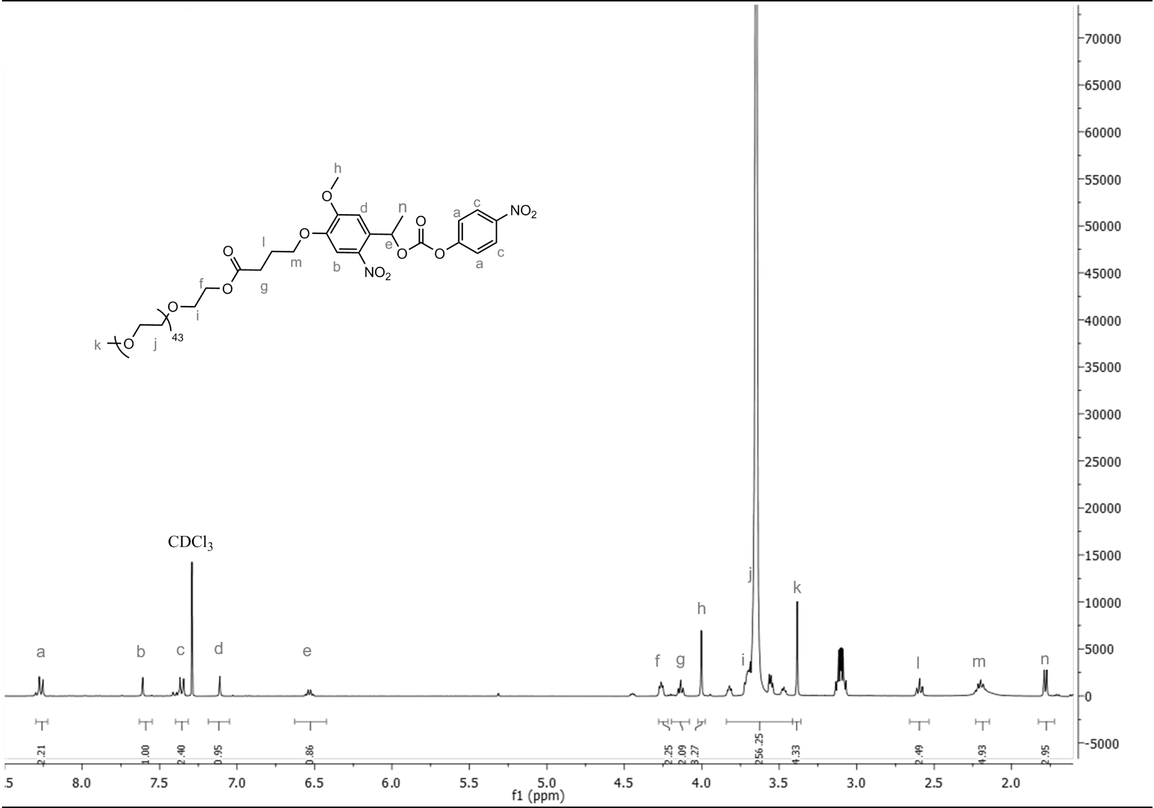

Figure S1. ^{1}H-NMR of **3**.

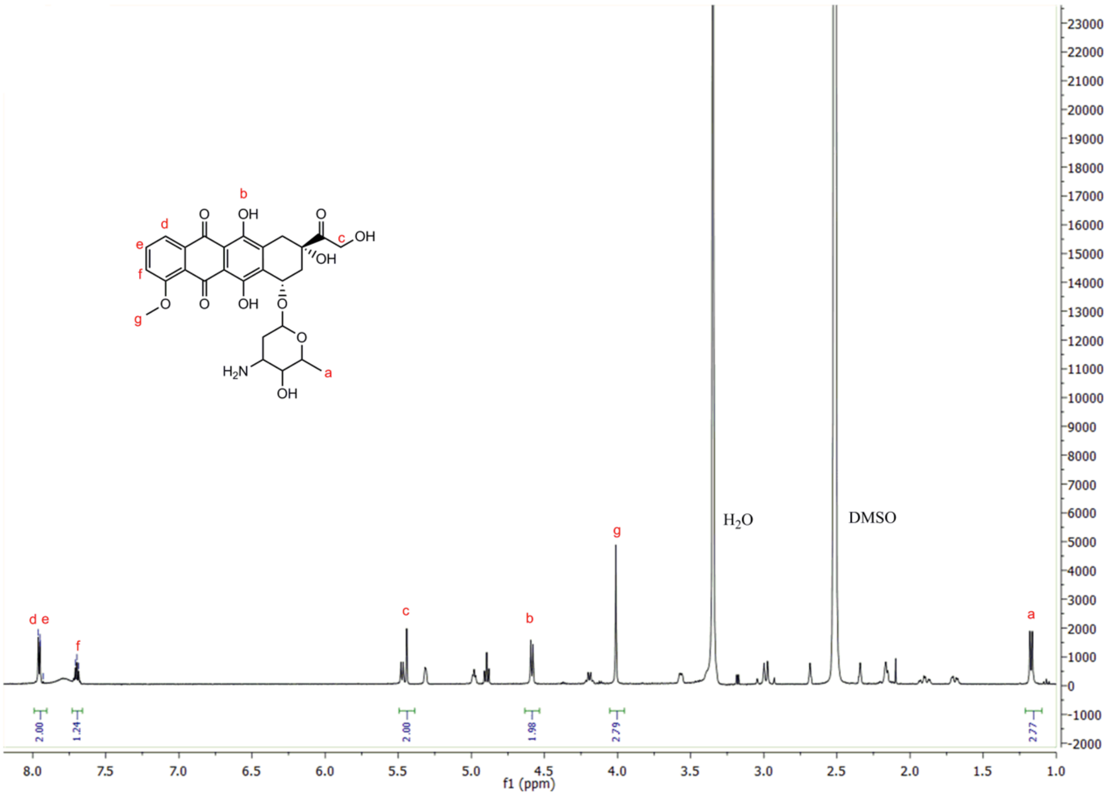

Figure S2. ^{1}H-NMR of **Doxorubicin**.

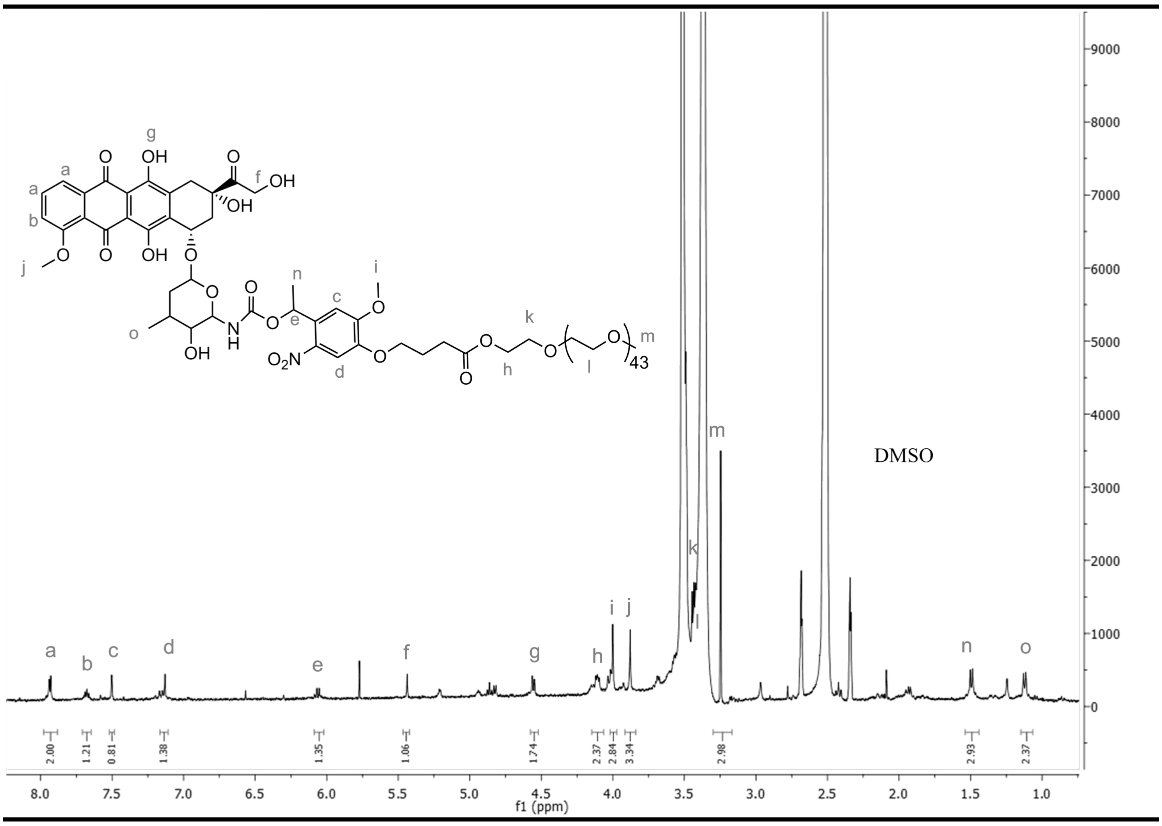

Figure S3. ^{1}H-NMR of **1**.

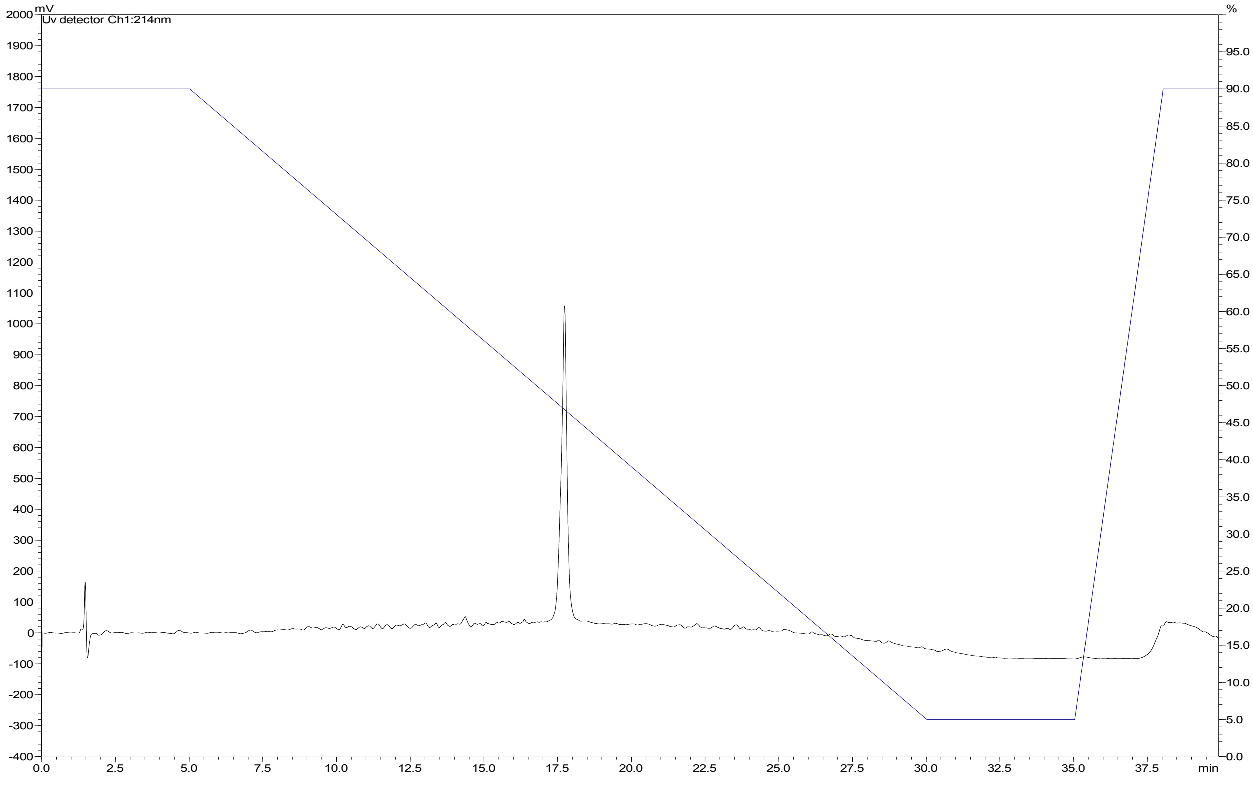

Figure S4. HPLC trace of **1**. Retention time – 17.8 min. UV detection – 214 nm.

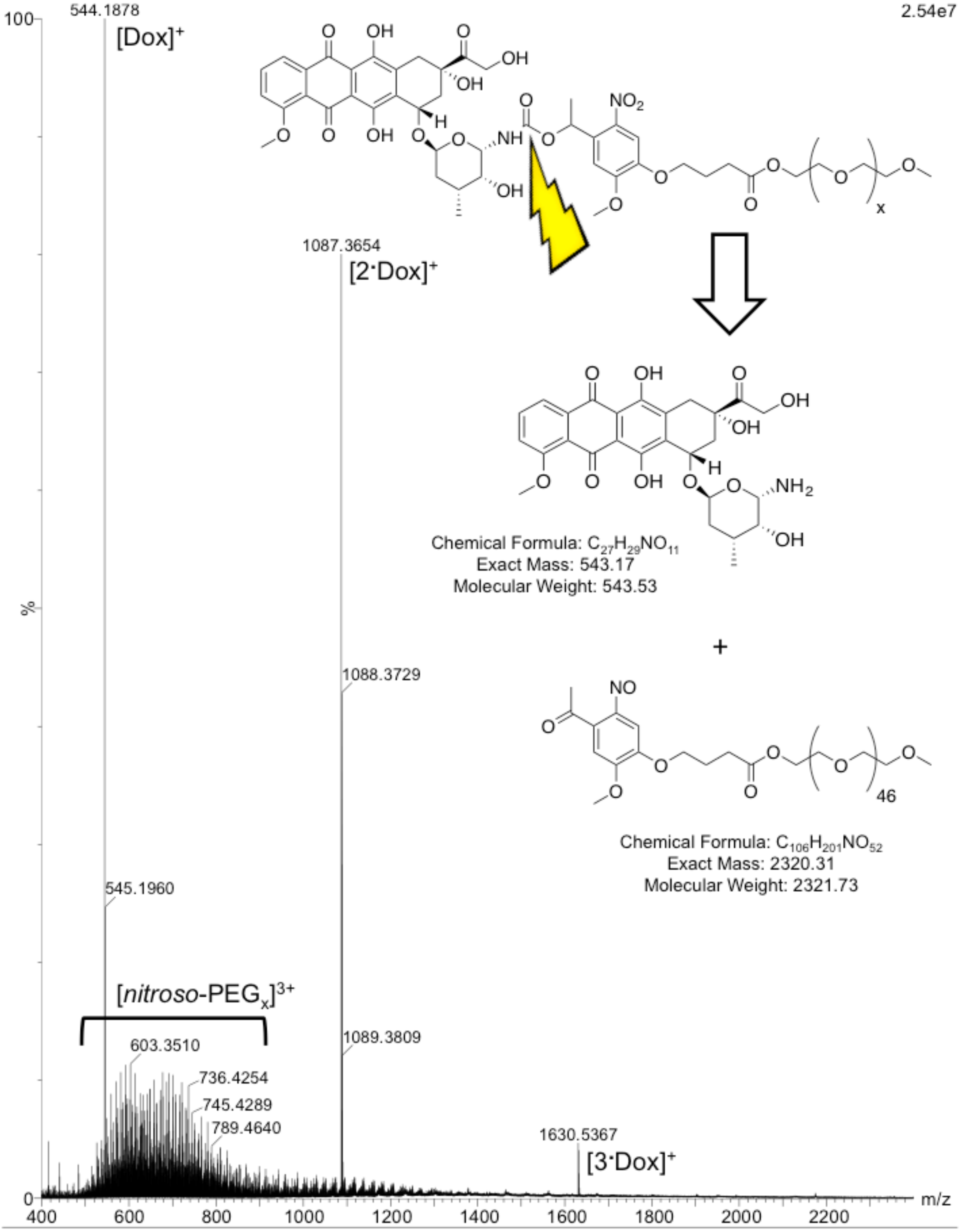

Figure S5. ESI-MS spectra (raw data) following photolysis of **1** and showing the expected photoproducts – DOX and nitroso-benzyl-PEG$_{2000}$ – as the only significant species present. The presence of DOX clusters – $[2\cdot DOX]^+$ and $[3\cdot DOX]^+$ - in the raw spectra arise from 'soft' elctrospray ionization techniques.

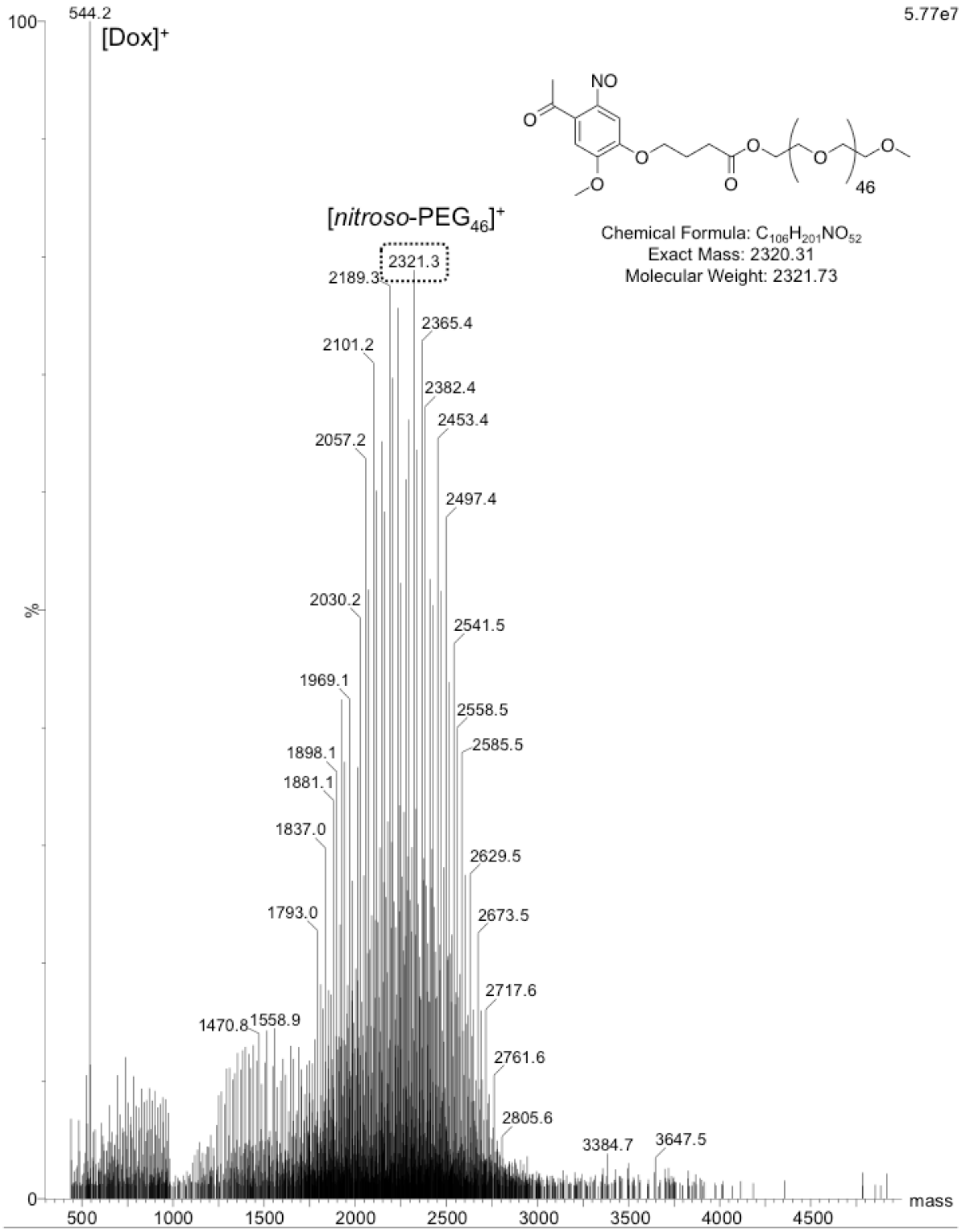

Figure S6. Deconvoluted (*software*: MaxEnt1) mass spectra of nitroso-PEG envelope signals.

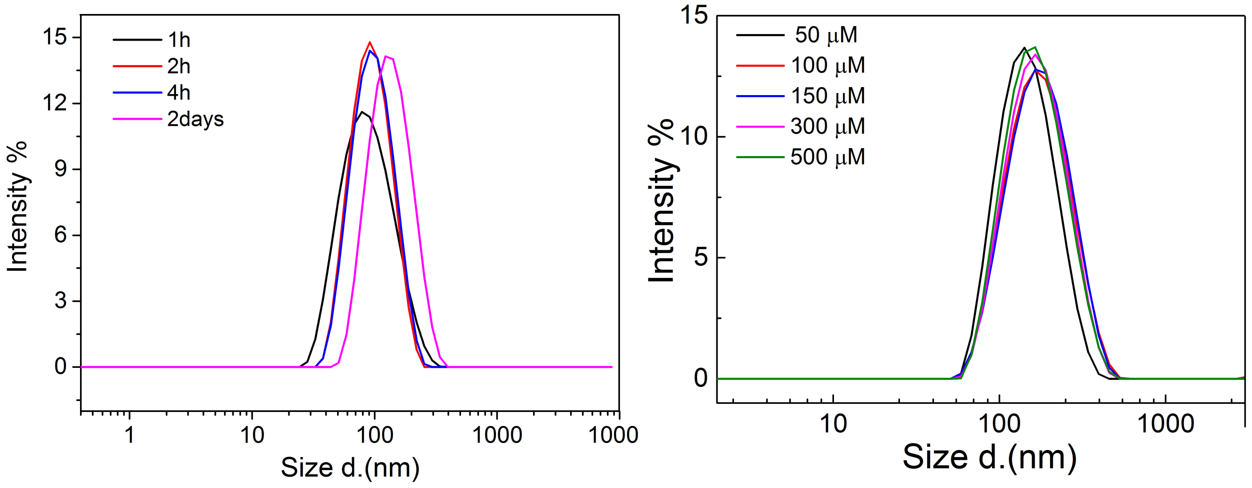

Figure S7. *(left)* Time course DLS size distributions of **1** (300µM in PBS) diluted (1:1) in DMEM+FCS. *(right)* DLS size distributions of **1** (varying concentrations) in PBS.

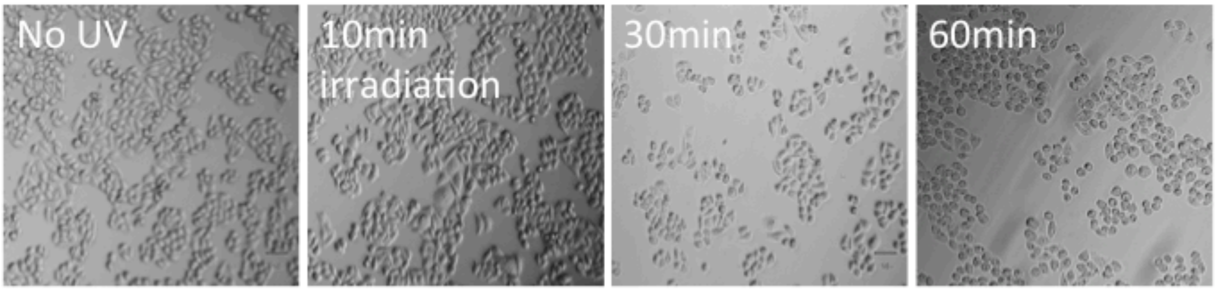

Figure S8. Cells (bright field) irradiated for varying times (UV-A, 365nm, 15-17 $mWcm^{-2}$) and imaged immediately. As UV-A irradiation times increase cells become smaller (shrinkage) and more rounded, hallmarks of the onset of UV-A induced apoptosis.[2]

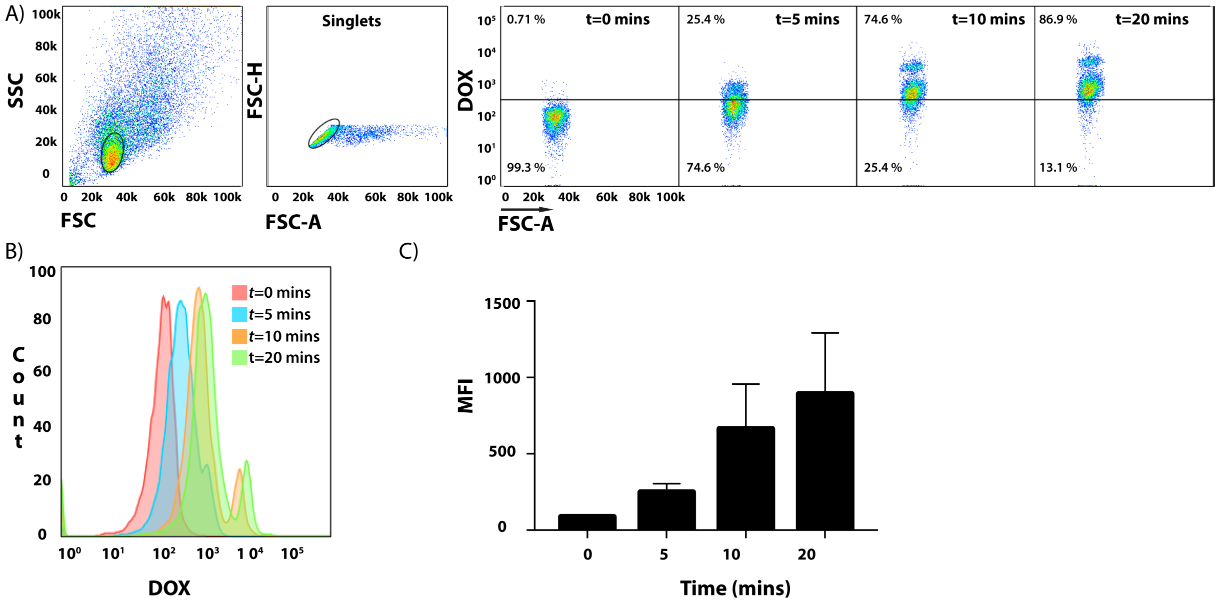

Figure S9. FACS analysis showing increased uptake of DOX (released from a solution of **1** (300µM in PBS)) by HeLa cells with increasing irradiation times. A) Dot plots of HeLa cells after *t*=0 ,5, 10 and 20 mins of irradiation; cell population was gated based on FSC-A vs SSC-A (cell doublets were gated out using FSC-A vs FSC-H). B) Histograms of HeLa cells after *t*=0 mins (pink) *t*=5 mins (blue), *t*=10 mins (orange) and *t*=20 mins (green) irradiation. C) Mean Fluorescence Intensity (MFI) of HeLa cells after different irradiation times. Error bars ± SD.

References

[1] Kong, L.; Askes, S. H. C.; Bonnet, S.; Kros, A.; Campbell, F. *Angew. Chem. Int. Ed.* **2016**, 55, 1396 –1400.

[2] Elmore, S. *Toxicol. Pathol.* **2007**, 35, 495–516.
